# ADDENDUM: Disruption of the striated muscle glycogen-targeting subunit of protein phosphatase 1: influence of the genetic background

**DOI:** 10.1530/JME-07-0120e

**Published:** 2025-03-18

**Authors:** James Paterson, Ian R Kelsall, Patricia T W Cohen

**Affiliations:** Medical Research Council Protein Phosphorylation Unit, College of Life Sciences, Sir James Black Centre, University of Dundee, Dundee, UK

The authors and journal apologise for an omission in the above paper, which appeared in volume 40 part 2, pages 47–59 . The error relates to Fig. 1A, given on page 51, and Fig. 3C, given on page 53.

The University of Dundee School of Life Sciences Research Integrity Group was informed of a potential duplication of data in the published work. Upon investigation, the authors confirm that the immunoblots in Fig. 1A were reused as part of Fig. 3C. However, in this case, duplication of these data was not incorrect. The rationale for this conclusion is as follows: the purpose of Fig. 1A was to confirm that the G_M_ subunit of protein phosphatase 1 was knocked out, as it was in Fig. 3C. The authors therefore assert that this duplication does not affect the data or their interpretation in any way. The authors accept that the duplication of data should have been clearly explained in the accompanying text and legends.

The amended legend to Fig. 3 is given in full below:

**Figure 3** (A) Glycogen content in skeletal muscle of C57BL/6 G_M_^+/+^ and lean C57BL/6 G_M_^−/−^ mice fasted overnight. Glycogen concentration is expressed in micromoles of glycosyl units per gram of muscle (wet weight). Results are expressed as mean ± SEM for 6 G_M_^+/+^ and 11 G_M_^−/−^ animals, and the difference between G_M_^+/+^ versus G_M_^−/−^ is statistically significant. *P < 0.001. (B) Left panel: phosphorylase kinase activity at pH 8.6 (in the presence of 40 μM Ca^2+^) in skeletal muscle lysates from wild-type ICR and PhK-deficient ICR/I mice. Results are expressed as mean ± SEM for three PhK^+/+^ and three PhK^−/−^ mice. Right panel: PhK activity in skeletal muscle lysates from C57BL/6 G_M_^+/+^ and lean C57BL/6 G_M_^−/−^ mice. Assays were performed in the presence of 40 μM Ca^2+^ at pH 6.8 (near physiological pH, open bars) and pH 8.6 (solid bars). Results are expressed as mean ± SEM for three to five animals of each genotype. *The difference in pH 6.8 PhK activities of C57BL/6 G_M_^+/+^ and lean C57BL/6 C57BL/6 G_M_^−/−^ mice was statistically significant, *P* < 0.05. All assays were performed in triplicate. Control values (1–2 mU/mg) using preimmune IgG in place of anti-PhK antibody were subtracted from the calculated activities. (C) Proteins in the PKB insulin signalling pathway in C57BL/6 G_M_^+/+^ and lean C57BL/6 C57BL/6 G_M_^−/−^ skeletal muscle lysates. The same representative immunoblot as given in Fig. 1A was used for G_M_ and PP1β in G_M_^+/+^ and G_M_^−/−^ mice. Mice were fasted overnight before injection of a bolus of either saline or insulin. Immunoblotting was performed using the indicated antibodies.

